# New Knowledge About Tissue Engineering Under Microgravity Conditions in Space and on Earth

**DOI:** 10.3390/ijms27010341

**Published:** 2025-12-28

**Authors:** Markus Wehland, Thomas J. Corydon, Luis Fernando González-Torres, Fatima Abdelfattah, Jayashree Sahana, Herbert Schulz, Ashwini Mushunuri, Hanna Burenkova, Simon L. Wuest, Marcus Krüger, Armin Kraus, Daniela Grimm

**Affiliations:** 1 Department of Microgravity and Translational Regenerative Medicine, Otto von Guericke University, 39106 Magdeburg, Germany; markus.wehland@med.ovgu.de (M.W.); luis.gonzaleztorres@med.ovgu.de (L.F.G.-T.); fatima.abdelfattah@med.ovgu.de (F.A.); herbert.schulz@med.ovgu.de (H.S.); ashwini.mushunuri@th-brandenburg.de (A.M.); hanna.burenkova@med.ovgu.de (H.B.);; 2 Clinic for Plastic, Aesthetic and Hand Surgery, Medical Faculty, University Hospital Magdeburg, Otto von Guericke University, Leipziger Straße 44, 39120 Magdeburg, Germany; armin.kraus@med.ovgu.de; 3 Research Group “Magdeburger Arbeitsgemeinschaft für Forschung unter Raumfahrt- und Schwerelosigkeitsbedingungen” (MARS), Otto von Guericke University, 39106 Magdeburg, Germany; 4 Department of Biomedicine, Aarhus University, 8000 Aarhus, Denmark; corydon@biomed.au.dk (T.J.C.); jaysaha@biomed.au.dk (J.S.); 5 Department of Ophthalmology, Aarhus University Hospital, 8200 Aarhus, Denmark; 6 Department of Engineering, Brandenburg University of Applied Sciences, 14770 Brandenburg an der Havel, Germany; 7 Space Biology Group, Institute of Medical Engineering, Lucerne University of Applied Sciences and Arts, 6052 Hergiswil, Switzerland; simon.wueest@hslu.ch

**Keywords:** microgravity, tissue engineering, spheroids, organoids, cancer, stem cells

## Abstract

Microgravity (µ*g*)-generated three-dimensional (3D) multicellular aggregates can serve as models of tissue and disease development. They are relevant in the fields of cancer and in vitro metastasis or regenerative medicine (tissue engineering). Driven by the 3R concept—replacement, reduction, and refinement of animal testing—µ*g*-exposure of human cells represents a new alternative method that avoids animal experiments entirely. New Approach Methodologies (NAMs) are used in biomedical research, pharmacology, toxicology, cancer research, radiotherapy, and translational regenerative medicine. Various types of human cells grow as 3D spheroids or organoids when exposed to µ*g*-conditions provided by µ*g* simulating instruments on Earth. Examples for such µ*g*-simulators are the Rotating Wall Vessel, the Random Positioning Machine, and the 2D or 3D clinostat. This review summarizes the most recent literature focusing on µ*g*-engineered tissues. We are discussing all reports examining different tumor cell types from breast, lung, thyroid, prostate, and gastrointestinal cancers. Moreover, we are focusing on µ*g*-generated spheroids and organoids derived from healthy cells like chondrocytes, stem cells, bone cells, endothelial cells, and cardiovascular cells. The obtained data from NAMs and µ*g*-experiments clearly imply that they can support translational medicine on Earth.

## 1. Introduction

Although the term “tissue engineering” had already sporadically been mentioned since the late 1930s and 1940s as a more theoretical concept [1,2], it was not until 1988, at the National Science Foundation Forum on Issues, Expectations, and Prospects for Emerging Technology Initiation, that it was formally given its current distinction, later refined by Langer and Vacanti as “an interdisciplinary field that applies the principles of engineering and the life sciences toward the development of biological substitutes that restore, maintain, or improve tissue function” utilizing isolated cells or cell substitutes, tissue-inducing substances, or cells placed on or within matrices [3]. Since then, the field has seen a surge in research activity, growing from 235 publications in 1993 to 21,799 reports in 2024. These numbers reflect the increasing importance the field has gained over the years, driven by the development of more sophisticated cell culture techniques, novel biocompatible materials and, especially in recent years, the emergence and widespread establishment and rapid refinement of 3D printing techniques.

Due to our increasingly ageing societies and generally rising life expectancy, we are faced with a rise in degenerative diseases such as osteoarthritis or osteoporosis, and also with conditions such as organ failure or injuries caused by loss of muscular strength and dexterity like fractured bones. Therefore, going forward, approaches to tissue and organ replacement therapy will gain even more importance. In 2023, over 170,00 solid organ transplants had been performed worldwide. Despite growing awareness and an increasing percentage of organ donors, these numbers only cover about 10% of the global need, as estimated by the 2023 International Report on Organ Donation and Transplantation Activities conducted by The Global Observatory on Donation and Transplantation (available from https://www.transplant-observatory.org/wp-content/uploads/2025/02/2023-data-global-report-20022025.pdf, last accessed on 6 December 2025). Tissue engineering might contribute considerably to filling this need in the future.

**Figure 1:**
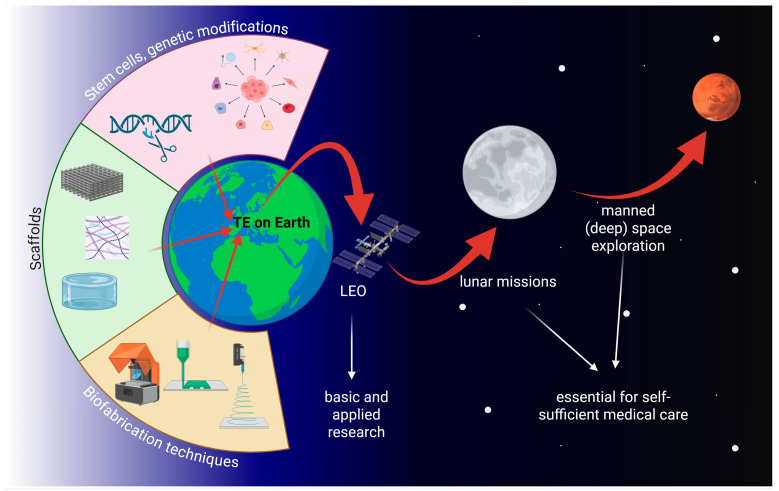
The three cornerstones of tissue engineering and its relevance for future manned space exploration. TE: tissue engineering; LEO: low Earth orbit. Created in BioRender. Wehland, M. (2026) https://BioRender.com/8uaw9gg, accessed on 7 December 2025.

While manned space research over the last decades was mainly confined to low Earth orbit on board the Space Shuttle or the Mir space station and the international space station (ISS), space agencies like NASA or the European Space Agency (ESA) have recently shifted their strategies towards deeper-space exploration of the Moon, Mars, and possibly beyond. At a certain distance from Earth, spacecrafts or colonies will have to be self-sufficient. As, due to the nature of this venture, all crew members play an important role in the missions and organ donors will not be available, tissue engineering might be the only viable option for emergency tissue or organ replacement. It is therefore important to explore tissue engineering under µ*g* and/or altered gravity conditions (Figure 1).

The principal aim of this concise review is to give an overview of the latest publications (2019–2025) regarding NAMs and tissue engineering approaches for healthy tissues such as cartilage, bone, endothelium, or heart, as well as spheroid formation of malignant tumors in both simulated (s-) and real (r-) µ*g*.

## 2. Microgravity Platforms

**Figure 2:**
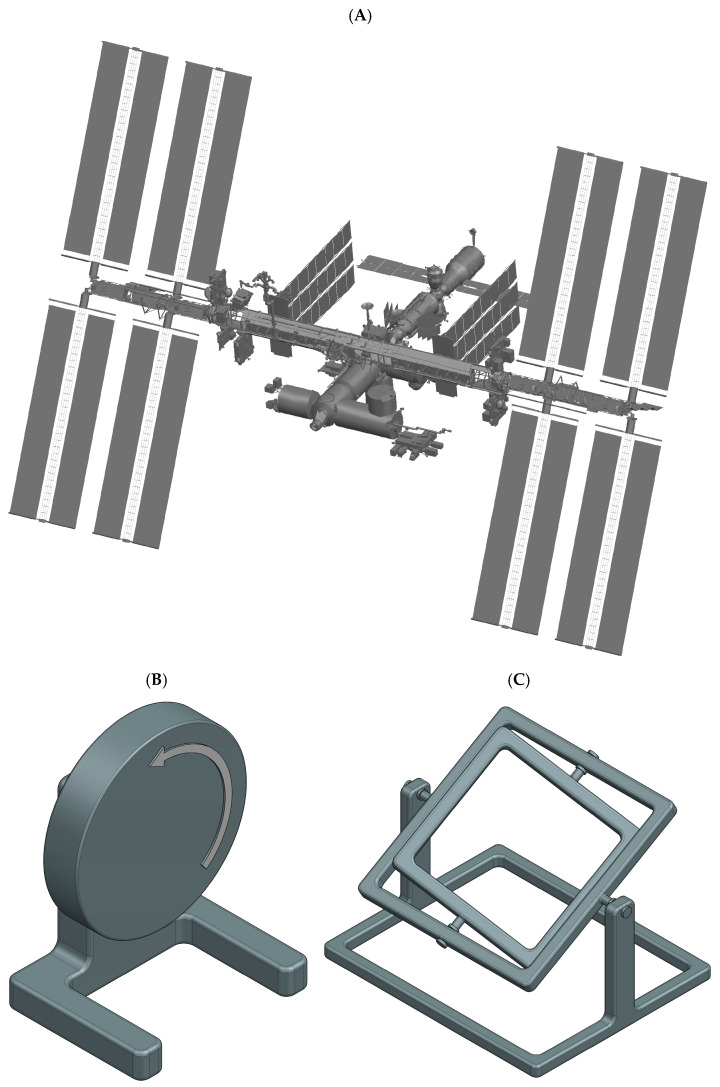
Schematic illustrations of µ*g*-platforms for tissue engineering applications. Space stations (**A**) provide continuous r-µ*g* in orbit. Clinostats (**B**) and the RPM machine (**C**) provide easily accessible low-cost s-µ*g*. Clinostats (**B**) and the related RWV rotate the sample about a single horizontal axis. RPMs (**C**), in contrast, rotate the samples continuously about two perpendicular axes.

Microgravity (µ*g*, also referred to as weightlessness) is typically created using dedicated µ*g*-platforms or alternatively by ground-based µ*g*-simulation methodologies (Figure 2). In real (and measurable) µ*g*, the samples are essentially in a state of free fall [4]. Short-term µ*g*-periods are achieved with drop towers (seconds) [5,6], parabolic flights (ca. 22 s) [7,8], and sounding rockets (minutes) [9]. However, tissue engineering applications require continuous µ*g*-conditions for days or even several weeks. Such long periods can only be achieved with orbiting platforms such as uncrewed return satellites [10,11] or crewed space stations.

### 2.1. Space Stations

Space stations, such as the ISS [12,13] or the Chinese Tiangong space station [14,15], provide well controlled, habitable environments (Figure 2A). The cabin is pressurized with a terrestrial atmosphere, and the temperature is maintained at room temperature (ca. 22 °C). Tissue engineering experiments are often integrated into existing facilities with standardized interfaces. The combination of a well-regulated environment on the space station and permanent in-orbit facilities makes hardware requirements less demanding compared to other space-flown platforms. In addition, the ISS offers specialized cold stowage systems (e.g., GLACIER and MELFI) to allow sample preservation prior to deployment or storage of fixed samples until their return to Earth [12]. Furthermore, crewed stations provide the key advantage of astronaut involvement for deploying experiments, handling samples, or troubleshooting in case of unexpected malfunctions. Nonetheless, as crew time is limited, experiments must be highly automated or capable of remote operation. A further caveat is the increased radiation present in orbit. Tissue engineering samples are typically delivered to the station and later retrieved as part of scheduled resupply missions. Upload cargo is launched aboard large rocket systems, allowing them to carry multiple tons of payload into orbit. These launches subject samples to moderate mechanical stresses (ca. 4–6 *g* linear acceleration and vibrations). Some launch vehicles even provide warm, cold, frozen, or powered sample uploads. However, one significant drawback is the risk of launch delays or cancellations. This poses challenges for live samples, where strict experimental timelines are critical and delays may compromise biological outcomes.

### 2.2. µg-Simulation Devices

Because space-based experiments are costly and involve long turnaround times, a variety of ground-based µ*g*-simulation devices are utilized [16,17]. It is important to emphasize that these devices do not generate a genuine µ*g*-environment in a physical sense. Nevertheless, they serve as valuable tools for conducting pre- and post-flight studies as well as ground tests during hardware development. While certain space experiments have been successfully reproduced using µ*g*-simulators, the results obtained from such platforms should always be interpreted with caution. Potential confounding factors may obscure or compromise a true µ*g* effect [18,19].

The most widely used methods for simulating µ*g* are based on rotation. Owing to their compact size and ease of operation, rotational devices have become standard equipment in many specialized laboratories. For life science experiments, it is generally assumed that organisms (e.g., cells or tissues) require a minimum period to sense and adapt to Earth’s gravity vector. By rotating samples at a rate faster than the biological process under investigation, the gravitational stimulus is effectively averaged out, creating a µ*g*-like condition. However, the rotation must be held at a minimum, as this can introduce additional mechanical stresses, such as centrifugal forces or shear stresses [20,21].

- Clinostats and Rotating Wall Vessels:

Clinostats [22,23] and the related Rotating Wall Vessels (RWVs) [24,25] rotate samples around a single horizontal axis. RWVs are typically used to suspended samples, with the rotation speed carefully adjusted to maintain suspension and prevent sedimentation. Compared to clinostats, RWVs usually rotate more slowly and feature larger-diameter and larger culture chambers volumes. Clinostats, on the other hand, can be used with both suspended and adherent samples. For suspended cultures, the rotation must likewise prevent sedimentation, allowing the sample to experience a continuous “free-fall”. In fast-rotating clinostats, speeds can reach up to 100 rpm, which requires the use of small-diameter culture chambers (such as serological pipettes) to minimize centrifugal forces. Both clinostats and RWVs share the advantage that, after an initial start-up phase, fluid movement within the chamber becomes minimal, thereby limiting shear stress on the samples [26].

- Random Positioning Machine:

Clinostat effectiveness is restricted to relatively small samples, typically not larger than a few millimeters. To overcome this limitation, the Random Positioning Machine (RPM) was developed [20,21]. The RPM consists of a gimbal-mounted platform that rotates samples continuously around two perpendicular axes in random directions and at variable speeds. With low rotation rates (around 10 rpm), centrifugal forces remain minimal (on the order of a few 0.01 *g*), even several centimeters away from the center of rotation [20]. This makes the RPM particularly suitable for larger specimens, such as biological tissues, organoids, or small organs. However, the dual-axis random rotation also generates complex fluid dynamics within liquid-filled culture chambers, exposing samples to additional shear stresses [27,28].

In summary, both r-and s-µ*g* platforms (Table 1) have proven to be invaluable tools for a wide range of tissue engineering applications. Space stations provide continuous, high-quality µ*g* and valuable facilities already implemented in orbit. In contrast, ground-based µ*g*-simulation devices are cost-effective and straightforward to use and are valuable complementary tools for tissue engineering experiments and applications in orbit.

## 3. Methods for Tissue Engineering

### 3.1. Cells

Although they already have the correct phenotype for the desired tissue, terminally differentiated cells are rarely used for tissue engineering, as they are difficult to propagate over a longer period and prone to dedifferentiation ex vivo. Therefore, various types of stem cells are generally preferred. They comprise totipotent embryonic stem cells, pluripotent or multipotent fetus-derived stem cells, and adult stem cells, as well as induced pluripotent stem cells (iPSCs), which are generated by reprogramming mature somatic cells (reviewed in [29]). Currently, mesenchymal stem cells are most widely used for tissue engineering, as they can be isolated relatively easily from liposuction material and have been found to induce virtually no immune response when transplanted into allogeneic recipients [30].

Immortalized tumor cells for the study of 3D cancer growth, on the other hand, have proven to be less difficult to handle. Due to their malignant nature, they can be propagated almost indefinitely and usually show robust growth rates (reviewed in [31]).

### 3.2. Scaffolds

When cells are cultured in vitro, they usually grow in a monocellular 2D-layer on the culture substrate until confluence. 3D-growth does not occur spontaneously. The aim of tissue engineering is the generation of replacement tissues, which need to have a certain three-dimensional shape to fit the lesion. It is therefore often necessary to use a scaffold, a support structure that provides a surface for the cells to colonize and determines the overall 3D shape of the resulting tissue. Scaffold materials comprise biopolymers such as collagen [32], gelatin [33], hyaluronic acid [34], alginate [35], or decellularized extracellular matrix (ECM) [36]; synthetic polymers like poly(lactic acid) [37], poly(lactic-co-glycolic acid) [38] or polyethylene glycol hydrogels [39]; as well as inorganic bioceramics such as hydroxyapatite or bioactive glass [40].

### 3.3. Scaffold Biofabrication

In tissue engineering, the most widely used biofabrication techniques are electrospinning, light-based stereolithography, and inkjet- or extrusion-based 3D printing.

Electrospinning yields fibrous scaffolds. It is relatively easy to set up, inexpensive, and is used for skin grafts or other cases where a more planar scaffold is necessary. During the process, a polymer solution in a syringe reservoir is held on a needle tip by its surface tension. Then, a high voltage is applied between the needle and a collector plate (or rotating drum) until the charge repulsion in the polymer overcomes the surface tension and a jet develops toward the collector. During the time of travel between reservoir and collector, the solvent evaporates and leaves a polymer fiber on the collector. In the case of a rolling setup, sheets of material composed of very uniform fibers can be produced [41].

Stereolithography uses photosensitive biopolymers, either already loaded with cells or pure. The initially liquid polymers are selectively polymerized via illumination with ultraviolet light using a digital micromirror array device to form optical pattern. Complex 3D scaffolds are created by repetitively adding polymer, layer by layer. This technique allows the generation of very fine structures but is somewhat limited by the polymer choice [42,43].

Extrusion-based 3D bioprinting, where a biomaterial is delivered by a piston-driven or hydraulic pump and extruded as a continuous cylindrical filament that can be layered on top of itself, has seen rapid growth over the last few years. The technique is relatively versatile, as a multitude of bioinks can be used, and so far, it is still the best suited for the reproduction of human anatomical shapes [44,45].

### 3.4. Scaffold-Free Tissue Engineering

While scaffolds offer the highest versatility in determining the final 3D shape of the tissue construct, they might be problematic in certain scenarios, as they potentially alter the physical properties of the tissue (elasticity, etc.), and for some materials, no long-term data are available yet. Therefore, scaffold-free tissue engineering techniques are an attractive alternative. They are all based on the principle of preventing cell adhesion to a surface, allowing cell–cell contact only. This can be achieved through constant stirring of the cell suspension (spinner culture), culturing cells in an agarose-coated microplate (liquid overlay), in an upside down hanging medium drop (hanging drop), or in a continuously rotating bioreactor (RWV, RPM, 2D and 3D clinostat, ClinoStar by Celvivo, Odense, Denmark). The major drawback of these techniques is their limited diversity of producible shapes, as they mostly generate spheroids/organoids (reviewed in [46]).

## 4. Results from Tissue Engineering Studies Performed in Space or on Earth

### 4.1. Cartilage

That the health and maintenance of articular cartilage is highly dependent on mechanical loading has already been established through various studies [47,48]. The reduced mechanical loading during long-term and short-term spaceflights can cause degeneration of articular cartilage, leading to bone loss and osteoarthritis. One step forward is to develop new tissue-engineering strategies for cartilage regeneration.

Steinwerth et al. proposed a method by engineering a dense 3D cartilage-like tissue without the addition of scaffolds or any other artificial materials in a s-µ*g*-condition [49]. This was realized by exposing primary human chondrocytes as well as cells from the immortalized line C28/I2 to an RWV bioreactor for 14 days. As a result, the C28/I2 cell constructs reached diameters up to 5 mm, while primary chondrocyte spheroids reached up to 3 mm. The authors observed that C28/I2 spheroids showed cartilage-like morphology with collagen types I, II, and X, but they were less responsive to RWV-induced gene expression changes. In contrast, primary chondrocytes displayed a significant regulation of multiple cartilage and stress-related genes, proving RWV bioreactors can generate dense, scaffold-free 3D cartilage-like tissues [49].

Additionally, Wehland et al. [50] studied cartilage-like tissue production in vitro using human articular chondrocytes grown in DMEM/F-12 medium exposed to s-µ*g* using an RPM. The results demonstrated the formation of scaffold-free 3D spheroids after 5 days, and by day 28, they reached sizes up to 2 mm in diameter. The addition of cartilage growth medium accelerated similar tissue formation at 14 days. Spheroids produced in both media exhibited cartilage-like morphology and aggrecan expression. Under RPM conditions, vimentin staining showed progressive filament clustering and mesh formation over 7–28 days. These spheroids were able to form a confluent monolayer when returned to 1 *g*, making them suitable for joint transplantation and cellular cartilage regeneration methodologies in trauma and OA therapy [50].

Furthermore, Aissiou et al. developed tissue-engineered cartilage through in vitro chondrogenesis using human bone marrow mesenchymal stem cells acquired from age-matched female and male donors to investigate the effect of r-µ*g* produced by parabolic flights. The resulting cartilage tissue from female donors exclusively showed upregulation of *WNT7B* and *WNT9A* genes from the Wnt-signaling pathway, which is associated with the development of OA [51]. A similar study was performed by Zhiyao et al. using primary meniscus fibrochondrocytes (MFCs) isolated from the inner avascular region of human menisci from both male and female donors. These cells were seeded into porous collagen scaffolds to generate 3D meniscus models and later subjected to both 1 *g* and s-µ*g* for 7 days. The results also showed sex-specific transcriptional responses such as an upregulation of the key OA markers *COL10A1*, *MMP13*, and *SPP1*, along with an increased cell proliferation in models derived from females [52]. Taken together, these studies suggest the possibility of a sex-dependent response of engineered cartilage tissues to µ*g* [51,52].

Another research group exposed engineered cartilage, developed by exposure to MFCs, to s-µ*g* and cyclic hydrostatic pressure (CHP). They observed that CHP and s-µ*g* had opposite effects on cartilage-related genes; CHP upregulated *ACAN* and *COL2A1* and suppressed the hypertrophy marker *COL10A1*, while s-µ*g* had the reverse effect. This study suggests that s-µ*g* may induce an OA-like profile, while CHP promotes chondrogenesis, and the combination of s-µ*g* and CHP could serve as a model to study the early molecular events of OA and potential drug-targetable pathways [53].

To summarize, exposure of chondrocytes with and without any scaffolds to s-µ*g* supports the formation of 3D cartilage tissues.

### 4.2. Bone and Muscle

**Figure 3:**
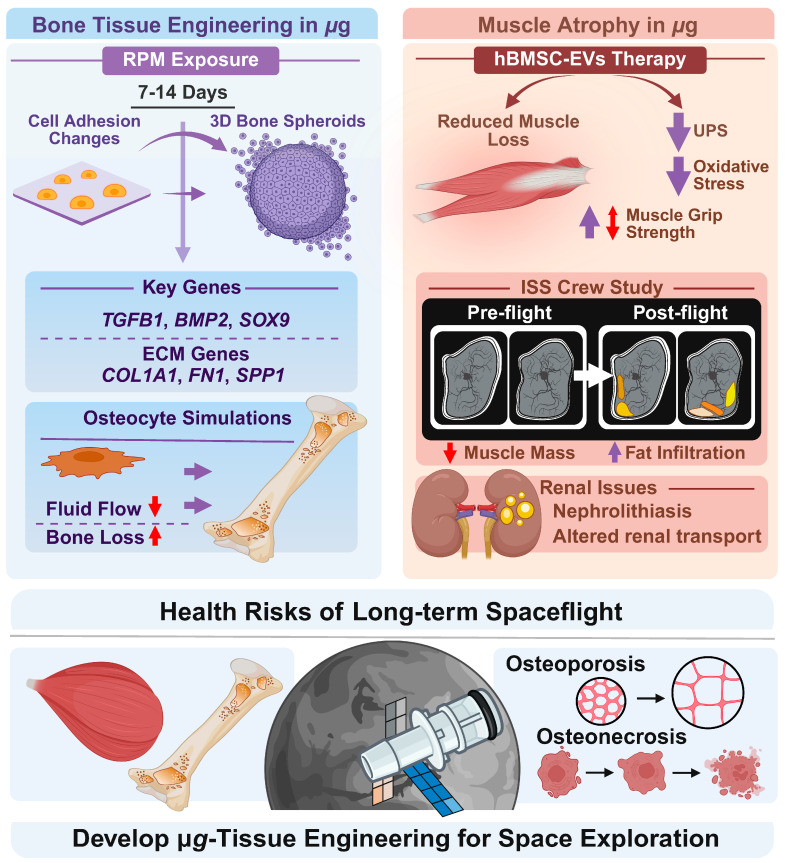
Illustration of bone tissue engineering [54], extracellular vesicles therapy [57], and health risks including muscle atrophy [55,56], osteopororosis, and osteonecrosis during spaceflights. Experiments in µ*g* can be used to develop tissue engineering for Space exploration. RPM-based studies show altered adhesion changes and formation of 3D bone spheroids. Bone loss is a consequence of exposure to µ*g*. Under normal and elevated gravity, osteocytes experience significant fluid pressure gradients, whereas µ*g* reduces fluid flow and shear stress across bone zones. ISS crew studies show muscle mass reduction and increased fat infiltration. Therapy based on hBMSC-EVs reduce muscle loss, muscle grip strength, UPS and oxidative stress. See text for further detail. Created in BioRender. Corydon Lab. (2026) https://BioRender.com/2y55at6, accessed on 22 December 2025.

Bones and tendons play essential roles in the human body’s structure and movement. Together, they form a critical part of the musculoskeletal system, allowing for stability, mobility, and physical function. In 2019, the effect of s-µ*g* on biological processes was studied by Mann and colleagues to aid the engineering of 3D bone structures [54]. In this study, human fetal osteoblast cells (hFOB 1.19) were exposed to an RPM for 7 and 14 days, resulting in significant alterations in cell adhesion, the cytoskeleton, ECM, and multicellular spheroid (MCS) formation. Notably, exposure to the RPM for 7 days markedly impacted the morphology, promoting detachment of adherent cells from the surface and facilitating their reorganization into 3D MCS structures. Moreover, following 14 days of RPM-exposure, the spheroids exhibited morphological characteristics indicative of bone-specific tissue organization (Figure 3).

These findings were augmented by gene expression analyses, revealing alterations across several functional categories. Key signaling or transcription factor molecules included *TGFB1* (transforming growth factor beta 1), *BMP2* (bone morphogenetic protein 2), and *SOX9* (SRY-box transcription factor 9). Cytoskeletal components such as *ACTB* (actin beta), *TUBB* (beta-tubulin), and *VIM* (vimentin) were also differentially expressed. In addition, ECM and adhesion-related genes—including *LAMA1* (laminin subunit alpha-1), *COL1A1* (collagen type I alpha 1), *SPP1* (secreted phosphoprotein 1), and *FN1* (fibronectin 1)—showed significant modulations [54].

In conclusion, this paper demonstrated that s-µ*g*-conditions offer a powerful strategy for bone tissue engineering while serving as a unique model to unravel the mechanisms of spaceflight-induced bone loss and pathologies, such as osteonecrosis and traumatic bone injury [54] (Figure 3).

It is well established that spaceflight induces skeletal muscle atrophy [55,56]. To further extend the understanding of the molecular mechanisms underlying muscle atrophy during offloading in µ*g*, Chang and coworkers investigated the impact of extracellular vesicles derived from human bone marrow mesenchymal stem cells (hBMSC-EVs) [57] (Figure 3).

Following isolation, hBMSC-EVs were co-cultured with nutrition-deprived mouse skeletal muscle myoblasts (C2C12) or intravenous injected in mice subjected to hindlimb unloading. Interestingly, hBMSC-EVs effectively reduced C2C12 myotube atrophy caused by nutritional deprivation. Additionally, the study demonstrated hBMSC-EVs-mediated down-regulation of protein levels associated with the ubiquitin proteasome system (UPS) and oxidative stress. In mice, hBMSC-EVs resulted in reversed reduction in soleus mass and grip strength caused by weightlessness. They also demonstrated the ability to inhibit protein degradation mediated by UPS and autophagy lysosome pathway. Finally, hBMSC-EVs prevented the phenotypic switch from slow oxidative to fast glycolytic muscle fibers [57].

In summary, hBMSC-EVs, through their anti-inflammatory and antioxidant properties, represent a novel and promising therapeutic approach for mitigating weightlessness-induced muscle atrophy, with potential implications for space medicine.

Poveda and coworkers also investigated the impact of µ*g* on muscle atrophy studying changes in muscle size, lean tissue, and fat infiltration in the thoracic and lumbar spine of nine ISS crewmembers using pre- and post-flight MRI [58]. Long-duration spaceflight reduces thoracolumbar muscle size and lean mass while increasing fat infiltration, particularly in the quadratus lumborum and transversospinalis. Exercise countermeasures show muscle-specific benefits: Treadmill training limits fat accumulation, cycle ergometry supports the psoas, and resistance exercise preserves lean mass across most spine muscles. These findings highlight the need for targeted strategies to prevent spaceflight-induced muscle atrophy and compositional changes.

To further foster research in tissue engineering under s-µ*g*-conditions, a novel scalable µ*g* simulator has been developed [59]. Improvements to the µ*g* simulator primarily involve optimizing movement-control algorithms and ensuring uniform treatment across all culture flasks under simulated microgravity. These enhancements are especially important for long-term cultures required in tissue engineering applications.

Prolonged spaceflights and advanced age may lead to irreversible osteoporosis. To study the mechanical response of osteocytes to the flow field under different gravity conditions, researchers have used numerical simulations applying a 3D axisymmetric fluid-solid coupling finite element model of a lacunar–canalicular system (LCS) of osteon with a two-stage pore structure [60,61]. The LCS is an essential microstructural basis for signaling and material transport in bone tissue. Under normal and elevated gravity, osteocytes experience significant fluid pressure gradients, whereas microgravity reduces fluid flow and shear stress across bone zones. This diminished mechanical and biochemical signaling, potentially leading to cell death, enhances osteoclast activity, and culminates in substantial bone loss [60]. Notably, moving loads at appropriate frequencies can enhance solute transport to middle and deep lacunae, preventing nutrient-deprivation-induced apoptosis of deep osteocytes [61]. These findings offer theoretical guidance for mitigating bone loss during long-duration spaceflight.

Another recent study investigated the mass transfer in bone LCS under different gravity fields [62]. Mass transfer in the natural LCS of bovine tibial cortical bone was examined using a sodium fluorescein tracer under varying gravity levels and pulsatile pressures (to simulate high intensity exercise in humans), revealing how different mechanical and gravitational conditions influence transport dynamics. High-intensity exercise and hypergravity enhance solute, nutrient, and signaling molecule transport in osteocytes, particularly to deep lacunae, promoting their activity. In contrast, microgravity may impair this transport, contributing to bone loss and osteoporosis [62].

Missions into Deep Space are planned for this decade. However, our knowledge of the health consequences of exposure to microgravity over year-long missions on indispensable visceral organs is scarce. Interestingly, a recent study by Siew and colleagues explored the impact of µ*g* using samples and datasets accessible from 11 spaceflight-exposed mice, 5 humans, 1 simulated microgravity rat, and 4 simulated galactic cosmic radiation (GCR)-exposed mice missions in an integrated pan-omic, physiological, and morphological study [63]. Notably, this comprehensive study demonstrated that spaceflight induces renal transporter dephosphorylation, suggesting that the elevated risk of nephrolithiasis in astronauts may arise, at least in part, from a primary renal mechanism rather than being solely a secondary consequence of bone loss. Without models of prolonged simultaneous exposure to µ*g* and full galactic cosmic radiation, current data offer only limited insight into the serious health risks of deep space travel. Comprehensive studies on the effects of spaceflight on visceral organs are critical to develop countermeasures and ensure the safety of future planetary missions [63]. More studies focusing on bone tissue engineering in µ*g* are necessary regarding the already started space exploration era.

### 4.3. Vessels

When exposed to a µ*g*-environment, some endothelial cells (ECs) are reported to spontaneously form MCS and tubular structures without scaffolds, resembling the early stages of blood vessel formation. This ability to induce complex physiological 3D structures could lead to the creation of functional tissues for medical applications, such as the production of biocompatible blood vessels for use in surgery [64]. In addition, other studies show that µ*g* can accelerate processes such as angiogenesis [65], which is advantageous for the vascularization of artificially produced tissue.

Endothelial cells are highly mechanosensitive and respond to alterations in their physical environment, including shear stress, tension, and gravity. Exposure to µ*g* disrupts the normal tension balance within the ECM and the cytoskeleton, promoting the spontaneous self-organization of ECs into 3D aggregates.

Although the influence of µ*g* on endothelial physiology has been extensively investigated, only a few studies in recent years have specifically addressed tissue engineering under µ*g*-conditions.

Krüger et al. reported that during the ESA-SPHEROIDS ISS-experiment, scaffold-free growth of human EA.hy926 cells into MCS and tubular structures was observed in space. This was the first experiment to demonstrate this growth behavior under r-µ*g*-conditions, which had previously only been observed on the RPM. Earlier preparatory studies had shown that the hybrid cell line EA.hy926 was the most suitable cell type for the space experiment [64].

In a follow-up study, Krüger at al. analyzed the molecular responses of EA.hy926 cells exposed to r- and s-µ*g*. The authors detected upregulation of cytokines and ECM proteins, such as VEGF, IL-6, IL-8 MCP-1, fibronectin, and collagen. Moreover, the authors also observed a reciprocal regulation between VE-cadherin (CDH5) and the pro-inflammatory cytokines IL-6 and IL-8. Under both r- and s-µ*g*, elevated levels of IL-6 and IL-8 coincided with decreased CDH5 expression, suggesting that these cytokines induce temporary junctional weakening and cell dissociation, allowing endothelial cells to migrate and rearrange in 3D tissues [66].

Jokšiene at al. [67] investigated EA.hy926 endothelial cells cultured on a 3D clinostat in media with low and high glucose concentrations. The cells maintained viability and structural integrity and formed stable spheroids. Under hyperglycemic conditions, both the number and size of aggregates increased, indicating that metabolic stress can enhance 3D morphogenesis [67].

To recap, culture of ECs under r- and s-µ*g* conditions revealed the formation of 3D tubular structures and MCS without any scaffolds. This was more pronounced under hyperglycemic conditions.

### 4.4. Heart

Results from recent reports show that engineering and culturing cardiac tissue under µ*g*- or spaceflight-conditions has potential beneficial effects on its structure and function. The alterations in mechanical stimuli derived from the µ*g*-environments cause unique effects on numerous molecular pathways that converge in structural and functional changes. These changes may be either beneficial or detrimental to engineered cardiac tissues depending on the type of cells, exposure duration, and µ*g*-platform. An example includes the results from a 30-day experiment on the ISS using neonatal and adult cardiac progenitor cells (CPCs) [68,69]. Compared to ground controls, adult CPCs on the ISS increased their *YAP1* expression after 12 days, followed by a regression by day 30. Additionally, RNA-seq analysis revealed 10,565 and 13,484 upregulated transcripts on the adult and neonatal populations, respectively, after 30 days on the ISS. Independent of age, biological processes related to proliferation, differentiation, heart development, oxidative stress protection, and focal adhesion were induced after 30 days on the ISS [69]. Following the same trend, adult CPCs under s-µ*g* on a 2D clinostat for 3 days showed an increased expression of *YAP1* and *SOD2*, a downstream target of YAP1, followed by a regression after 7 days. YAP1 is a known target of the Hippo signaling pathway, which regulates cell proliferation and survival, and its depletion is associated with apoptosis in cardiomyocytes [70]. Thus, increased expression of *YAP1* under s-µ*g* suggests an increase in proliferation and survival, which are desired attributes in cardiac tissue models.

Human-induced pluripotent stem cell-derived cardiomyocytes (HiPSC-CMs) have also been investigated under µ*g*- or spaceflight conditions and have produced promising results. 2D HiPSC-CMs cultured during 5.5 weeks on the ISS showed a retention of sarcomere structure, length, and regularity compared to ground controls, as well as similar beating rate, contraction and relaxation velocities, and Ca^2+^ transient amplitude [71]. Moreover, RNA sequencing revealed 3008 differentially expressed genes (DEGs) between flight and ground samples, among which hypertrophy-related genes (*TNNT2*, *TNNI1*, *MEF2D*, and *HDAC8*) were upregulated, and 1049 DEGs between post-flight and ground samples, indicating that HiPSC-CMs adopt a unique gene expression signature after spaceflight. Finally, pathways related to mitochondrial function and cardiac metabolism were significantly enriched, while motif enrichment analysis revealed motifs enriched by upregulated genes associated with transcription factors that regulate hypertrophic pathways. Nonetheless, increases in transient decay tau and standard deviation of beating intervals suggested decreased Ca^2+^ recycling rate and beating irregularity, respectively [71].

Additionally, results that promote cardiac tissue maturation parameters have been obtained from 3D-cultured HiPSC-CMs under µ*g*-conditions. S-µ*g* exposure via an RPM for 7 days resulted in increased Ca^2+^ transient amplitude, maximum rise slope, and maximum decay slope [72]. It also produced increases in nuclear diameter, z-disk length, sarcomere length, mitochondrial content, mitochondrial membrane potential, and mitochondrial function of HiPSC-CMs, all of which are usually increased in matured cardiomyocytes in comparison to fetal cardiomyocytes [73]. 3D HiPSC-CMs in the form of spheres were also cultured on the ISS for both 3 days and 3 weeks [74]. Increased gene expression related to cell cycle and proliferation (*CCND2*, *CCND1*, *TBX3*, *IGF2*) was reported for both time points, as well as an increase in sphere diameter and Ki-67 positive cells, indicating enhanced proliferation during spaceflight. Cardiac tissue maturation parameters were also enhanced, including more organized myofibrillar structures, enhanced Ca^2+^ transient kinetics, and increased expression of cardiac structure-related genes (*MYL2*, *TNNI3*, *TNNT2*, and *MYH7*). Moreover, RNA-seq of the 3-day samples detected 402 DEGs, of which 195 were upregulated and mostly related to cell cycle, proliferation, survival, and regeneration [74]. Meanwhile, RNA-seq for the 3-week samples detected 470 DEGs, of which 271 were upregulated and mostly involved in cell cycle, proliferation, survival, and cardiac differentiation [75]. Finally, a similar study also cultured HiPSC-CM spheres for 8 days on the ISS [76]. Results showed upregulation of proteins related to survival, proliferation, and metabolism (UGT2A3, EEF2K, KRT13, PRIM1); at the same time, genes related to mitochondria, metabolism, and cardiac development (*TNNT2*, *PLCB2*, *CPNE5*, *CDH17*, *SFRP5*, *PLA2G4F*, *GPAT2*) were also upregulated.

It must be stated that not all HiPSC-CMs models exposed to µ*g*-conditions result in enhanced cardiac tissue parameters. 3D HiPSC-CMs cultured within a decellularized porcine myocardial extracellular matrix scaffold with reduced graphene oxide were cultured on the ISS for 30 days [77]. Results from this automated heart-on-a-chip model showed decreased contractile twitch force and increased arrhythmic activity and structural damage in terms of shortened sarcomeres and fragmented mitochondria. Moreover, contractility-related genes (*MYL7*, *MYL3*, *MYH7B*, *TNNI3*, *RYR2*, *ATP2A2*, *TTN)* were downregulated, while inflammation and oxidative stress-related genes (*NOXO1*, *GGTLC3*, *IL18*, *TGFB2*) showed upregulation [77]. Another study cultured 2D HiPSC-CMs on the Chinese Space Station for 6 days and reported decreases in cardiomyocyte size, sarcomere length, cardiac troponin T content, ATP production, contractile function, and slower Ca^2+^ cycling [78]. These examples show that the enhancement of cardiac tissue maturation markers for HiPSC-CMs under spaceflight conditions is not always observed, and in some cases, it may be detrimental. Further research is necessary to elucidate the exact mechanisms and conditions under which µ*g* may be a beneficial factor in the maturation of cardiac tissue.

Similarly, 2D HiPSC-CMs cultured for 2 days under s-µ*g* on a 2D clinostat showed structural and functional changes, such as 3D spatial chromosome reorganization, formation of stress fibers and membrane caveolae, increased reactive oxygen species and senescence markers, altered Ca^2+^ transient parameters, reduced contraction and relaxation velocities, and impaired mitochondrial membrane potential and ATP production [79]. Additionally, RNA-seq revealed 825 DEGs compared to 1 *g* controls, of which 562 were upregulated. Gene Ontology (GO) enrichment and KEGG pathway analyses of the upregulated genes revealed enriched biological processes related to ECM organization, hypoxia, glycolytic metabolism, heart development, and Ca^2+^ homeostasis. Meanwhile, downregulated genes enriched biological processes related to ATP production and mitochondrial metabolism. Proteome-wide analysis also revealed 56 down- and 123 upregulated proteins, which were mostly associated with DNA damage and oxidative stress-induced senescence [79]. Overall, s-µ*g* caused stress-related changes after only 2 days of exposure, resulting in a senescence-like state for the HiPSC-CMs. Whether this state is reversible upon return to 1 *g*, or in response to anti-senescence compounds, remains to be investigated and may be an important contribution to space cardiovascular disease modelling.

Thus, µ*g*-conditions may be exploited for cardiac tissue maturation purposes on HiPSC-CMs and may help advance heart research and therapy developments. However, the potential beneficial results may be dependent on cardiac model, µ*g* platform, and exposure duration. One of the potential applications of matured HiPSC-CMs is in the field of cell therapy, and one of the big challenges of this field is the understanding and control of tissue maturation to generate differentiated cardiac cells with an adult phenotype. This adult phenotype is also crucial for drug screening and adult-onset disease modeling [73].

In the future, the use of µ*g* alone or in combination with other tissue maturation strategies may lead to the development of tissue models with an adult phenotype that produce clinically relevant results.

Table 2 provides a short summary of all µ*g* tissue engineering results with normal tissue cells.

### 4.5. Organoids and Multicellular Tumor Spheroids

**Figure 4:**
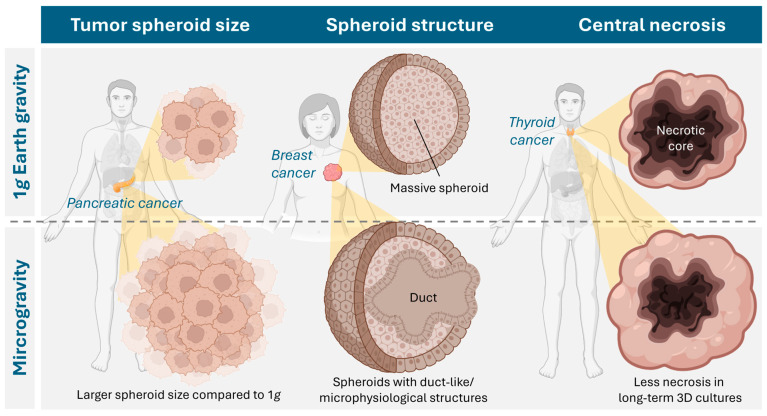
Cancer types studied in microgravity and described effects of microgravity culture on the properties of tumor spheroids. Created in BioRender. Krüger, M. (2026) https://BioRender.com/7lbteiv, accessed on 23 December 2025.

Cancer is one of the greatest disease burdens worldwide, with almost 20 million new cases and 10 million deaths in 2022, and the trend is rising [80]. The health and economic consequences are immense and represent a major obstacle to increasing life expectancy. At the tissue level, the native cancer microenvironment is highly dynamic and exhibits a distinct ECM, cell–cell, and cell–ECM interactions that vary depending on the stage of the disease and regulate cancer growth, angiogenesis, aggressiveness, invasion, and metastasis [81,82]. A complex interplay of different cells, such as non-cancerous fibroblasts, adipocytes, immune and vascular cells, as well as signaling molecules and mediators that contribute to the progression of the disease, complicates cancer treatment. 2D models fail to replicate the microenvironment and architecture of a tumor in vivo [83,84]. Animal models can overcome these limitations, but they are expensive, time-consuming, and often require complicated surgeries. Above all, however, due to the absence of human cells and their immune response, they cannot mimic human physiology, which means that preclinical findings cannot always be transferred. In addition, several ethical concerns regarding animal welfare have been raised, which is why the use of animal models must be handled rationally and carefully [85]. Recent developments in tissue engineering have demonstrated that 3D in vitro intermediate models (spheroids, organoids, tumoroids) can be developed that mimic realistic in vivo niches of cancer tissue, enabling a better understanding of the disease and the development of novel cancer treatment approaches (tumor engineering; Table 3) [86,87,88,89,90,91]. These 3D structures typically retain the genetic and epigenetic properties and morphology of their tumor of origin and can therefore be used to understand the underlying mechanisms of cancer initiation, progression, and metastasis in a more physiological setting. Furthermore, co-culture methods of tumoroids and cancer-associated cells can help to understand the interactions between a tumor and its tumor microenvironment [92,93]. While conventional platforms (hanging drop, spinner flask, agarose overlay, 3D bioprinting, organ-on-a-chip) are already being used extensively in cancer research, methods that utilize (simulated) microgravity are still in their infancy. Nevertheless, approaches to tumor engineering in microgravity benefit from the fact that 3D aggregates of cancer cells (Figure 4) are generally easier to form there than those of benign cells [94]. The following sections focus on findings relating to 3D cell culture of tumor cells in a microgravity environment.

### 4.6. Breast Cancer

Data published on the effects of µ*g* on breast cancer cells is based on experiments in r-µ*g*, such as sounding rocket and parabolic flights, and also on ground-based simulation devices. Concerning culture techniques, scaffold-associated and scaffold-free models have been published. The scaffold-free approach seems to be the more widely used. Regarding scaffold-based experiments, a recent study used spheroids created on spherical plates, then imbedded in hydrogels of various stiffnesses and exposed to s-µ*g* (2D-clinostat) for seven days [95]. This study reported that s-µ*g* could modulate and partially reverse the effects of a stiffer ECM on breast cancer cells (MCF-7 and MDA-MB-231). This shows a deep impact of s-µ*g* not only on cell–cell but also on cell–matrix interaction. When low-invasive (MCF-7) and highly invasive (MDA-MB-231) breast cancer cells were exposed to s-µ*g* for 14 days in a RPM, various effect occurred [96]. In these long term-experiments, it was observed that in MCS, the microenvironment was more like the real metastatic microtumor environment concerning ECM, cytoskeleton, cell morphology, and expression of pivotal genes of different signaling pathways than conventional 2D culture. The authors concluded that s-µ*g* is a versatile cell culture model for studying breast cancer cell behavior and drug testing.

Another study from the same group evaluated adhesion mechanisms of breast cancer spheroids from MCF-7 and MDA-MB-231cells under s-µ*g* [97]. Interestingly, the MDA-MB-231 cells had mostly detached from the cell culture flask after 24 h, while the MCF-7 cells remained mainly adherent under s-µ*g*. Gene expression analysis revealed various changes for both cell lines in adhesion-related genes. In MDA-MB-231 spheroids, *ITGB1*, *TLN1*, and *VCL* gene expression was downregulated, whereas in MCF-7 spheroids, when they had formed later, downregulation of *PXN*, *TLN*, and *CDH1* gene expression was observed. Gene expression and protein synthesis was also altered in a study by Strube et al., where breast adenocarcinoma cells (CRL2351) were exposed to s-µ*g* (RPM) for up to five days [98]. The findings on the gene expression and protein synthesis level indicated a more invasive and aggressive tumor phenotype. Particularly, *BRCA1* and *HER2*/ERBB1 were upregulated under s-µ*g*, with upregulation of *VIM*, *RHO*, and *MAPK1*. Corresponding protein synthesis was partially similar but in part contradictory to these gene upregulations. The same group found pronounced cell detachment and spheroid formation of CRL2351 cells after 24h of s-µ*g.* At this earlier time point, compared with the experiments described above, significant alterations of gene expression and protein synthesis already occurred [99]. According to the authors, this altered expression profile indicated a tendency towards modified adhesion properties, enhanced cell repair, and phenotype preservation.

A further study examined the release of exosomes (EVs) in MCF-7 cells [100] under s-µ*g* (RPM) after 5 and 10 days. An increase in the release of exosomes was observed compared to 1 *g*-conditions, and there was also an alteration in the distribution of exosome subpopulations. This shows that not only intracellular changes occur under s-µ*g*, but also that intercellular communication is profoundly affected by external forces. The release of exosomes under s-µ*g* was also evaluated in a study by Chen et al. [101]. Triple-negative MDA-MB-231 breast cancer cells were exposed to s-µ*g* (3D-clinostat) for two months. The authors found the number of EVs to be decreased under s-µ*g*, but their size was increased. Protein expression significantly changed in the EVs under s-µ*g*. Some proteins, such as monocarboxylate transporter 4 and major histocompatibility complex I, were upregulated, while others, such as long-chain-fatty-acid CoA ligase 4, guanine nucleotide-binding protein G(k) subunits alpha, and guanine nucleotide-binding protein G(q) subunits alpha, where downregulated. According to the authors, these results indicate decreased cell migration and proliferation and hint towards a less malignant phenotype.

Apoptosis is an issue that seems to be strongly influenced by gravitational forces. When apoptosis of somatic breast cells (MCF10A) and breast cancer cells (MCF-7) under s-µ*g* was compared, considerable differences were found [102]. MCF-7 cells showed a far higher apoptosis rate than MCF10A cells under s-µ*g* after 72 h. Particularly, apoptosis was highest in the spheroid section of the MCF-7 cells, whereas surface-bound cells showed a lower apoptosis rate. The authors found that both cell lines upregulated Akt- and ERK-dependent pathways to protect against apoptosis, but upregulation was less pronounced in cancer cells than in somatic breast cells.

Overall, both r- and s-µ*g* have a profound impact on various breast cancer cells lines regarding growth behavior, spheroid formation, morphology, gene expression, protein synthesis, and excretion.

### 4.7. Lung Cancer

Lung cancer cells have been shown to be profoundly influenced by r- and s-µ*g* in various recent studies. From a technical point of view, spheroid formation of lung cancer cells in rotating devices, such as 3D-clinostats, is well established. Optimal culture conditions, such as via addition of respective media supplements and several details of cell culture handling, however, are a requirement for a successful spheroid formation [103].

Baghoum et al. [104] exposed the lung cancer cell line A549 to s-µ*g* (2D-clinostat) for up to 72 h. Particularly, besides a decreased proliferation rate, there was increased expression of *FCGBP*, *BPIFB*, *F5*, *CST1*, and *CFB.* These results were interpreted as a reversion of epithelial-to-mesenchymal transition (EMT), which is generally associated with cancer progression. Further evaluation concerning gene expression associated with disease prognosis remained contradictory, as both genes of good prognosis, such as FCGBP, and genes of poor prognosis, such as *NOX1*, *GDA*, *TCN1,* and *BPIFA1* were upregulated. The same cell line (A549) was exposed to s-µ*g* (RPM) by Degan et al. for up to 48 h [105]. The authors found reduced cell proliferation, with most cells remaining in G1 and G2 phase. The number of polynucleated cells, which might function as blastomere-like stem cells, was strongly induced by s-µ*g*. Several microRNAs (miRNAs) associated with malignancy, cell cycle regulation, and apoptosis were downregulated, but others were upregulated under s-µ*g*. In addition, the migration behavior of A549 cells, as well as of H1703 cells, was altered under s-µ*g* [106]. In a scratch assay test, migration of both cell lines was considerably faster when conditioned under s-µ*g* in an RPM. In both cell lines, expression of genes related to invasiveness (*MMP-2*, *MMP-9*, *TIMP-1,* and *TIMP-2*) was upregulated under s-µ*g*. Protein expression of MMP-1 and MMP-2 was upregulated in A549 but not in the H1703 cells. This discrepancy shows again the profound cell-individual differences in the reaction of different cell lines to s-µ*g.*

A further study with squamous non-small-cell lung cancer cells (CRL-5889) exposed to an RPM for up to 96 h revealed various changes [107]. 3D spheroid formation occurred as early as after 24 h and became increasingly pronounced towards the last investigation point at 96 h. Cytoskeleton alignment changed from a longitudinal to a more spherical shape when compared to the 1 *g* controls. The apoptosis rate of the cells was significantly higher under s-µ*g* than under 1 *g*, both after 24 and after 96 h. There was an upregulation of several tumor suppressor genes (*TP53*, *PTEN*, *RB1*, and *CDKN2A*), and also of the oncogene *SOX2* compared to the 1 *g* control group. These effects were generally more pronounced in the cells remaining adherent than in the spheroids. Enhanced synthesis of the corresponding proteins could be detected in part, but they were generally not as distinct as on the gene level.

Some details about adhesion mechanisms in lung squamous cell carcinoma cells (H1703) and adenocarcinoma cells (Calu-3) under s-µ*g* were clarified in a study by Barkia et al. [108]. Both cell lines showed different abilities to form 3D spheroids. The H1703 cells showed a much stronger tendency to form spheroids than the Calu-3 cells under s-µ*g* after 72 h. The more detailed evaluation of the underlying mechanisms revealed a lower mucin-1 expression in the Calu-3 cells, which was further downregulated under s-µ*g*. Spheroid formation of Calu-3 cells was possible, but these spheroids were unstable due to an imbalance in adhesive (β_1_-integrin, E-cadherin) and anti-adhesive proteins (mucin-1). Calu-3 cells showed a higher expression of the estrogen receptor than H1703 cells, and consequently, addition of 17β-estradiol led to higher stability of spheroids with higher expression of E-cadherin in this cell line.

These results again show that spheroid formation under s-µ*g* is far more prevalent than the effect of mechanical disruption from surface adherence due to mechanical movement. It is a very cell-specific process with a complex interplay between intercellular communication, soluble factors, and likely several endocrine and paracrine factors. In lung cancer cells, s-µ*g* induces complex changes in morphology, apoptosis, gene, and protein expression. As all these data are acquired from experiments under s-µ*g*; further studies in r-µ*g* are needed to obtain further information about gene and protein expression, apoptosis, invasiveness, and malignancy in space.

### 4.8. Thyroid Cancer

With 586,000 cases worldwide, thyroid cancer ranked 9th in terms of incidence in 2020, with an incidence rate three times higher in women than in men [109]. The following chapter describes microgravity-assisted spheroid development in cell lines from three histological groups of thyroid cancer: follicular (FTC-133 and WRO cell lines), anaplastic (SNU-80 cell line), and papillary carcinoma (SNU-790 cell line).

#### 4.8.1. Follicular Thyroid Carcinoma

The synthetic glucocorticoid dexamethasone (DEX) suppresses spheroid formation under s-µ*g* in a culture of FTC-133 follicular thyroid cancer (FTC) cells in a dose-dependent manner. This was reported by Melnik and coworkers in 2020 [110] through RPM experiments of either 4 h or 3 d. Their results led to the assumption that the decisive role in FTC-133 spheroid formation was played by more complex Wnt/β-catenin and TGF-β related signaling pathways rather than NF-κB signaling. In their response to the article, Bevelacqua et al. [111] emphasized that RPM causes increased convection and shear forces compared to experiments under r-µ*g* conditions, as previously demonstrated by Wuest et al. [19]. These increased forces can have a lasting effect on the experimental results. In their response, Krüger and colleagues [112] pointed out that the intention of the initial study was not to simulate space conditions. They argued that shear stress also plays a role in carcinomas under physiological conditions in the human body, and that radiation acts as an additional stressor in r-µ*g* experiments. Two years later, Melnik et al. [113] presented a study focusing on the different response of DEX on benign thyroid cells and corresponding FTC cells exposed to an RPM for 3 d. They observed that in metastatic FTC-133 and WRO cells, DEX enhanced adhesion via increased tight junction (TJ) formation in conjunction with elevated expression of the TJ proteins claudin-1 and ZO-1. Low claudin-1 levels in particular play a role in the development of highly metastatic cancer cells and dedifferentiation. In 2023, Cortés-Sánchez et al. [114] conducted a 3 d RPM experiment using FTC-133 cells to demonstrate the mechanisms leading to cell detachment and the formation of multicellular tumor spheroids. First, the detachment of cells triggered by rotation is mainly caused by fluid flow and depends on cell density, the amount of matrix and adhesion proteins (adhesion forces), as well as physical shear forces (flow rate, bubbles, and surface conditions). Secondly, the efficiency of MCS-aggregation is mainly dependent on the prevention of sedimentation of the adherent cells.

In 2022, Melnik et al. [115] reported the effects of 5 and 10 d exposures of FTC-133 cells to r-µ*g*. The experiment was framed by the CellBox-2 Mission to the ISS. In contrast to the ground controls, the cells formed MCS with a mainly anti-proliferative response to µ*g* in space. The focal adhesion molecules vinculin (*VCN*) and paxillin (*PXN*), involved in actin-membrane attachment, were generally downregulated in the spaceflight samples. The epidermal growth factor receptor (*EGFR*) was significantly downregulated after 10 d in all spaceflight samples. In contrast, *VEGFD* gene expression was increased in the spaceflight samples, even stronger than in MCS, while the amount of secreted VEGFD was stable under all conditions. The r-µ*g VEGFD* gene regulation was taken as an indication of redifferentiation processes. The downregulation of the IL-8 cytokine coding gene (*CXCL8*) in samples exposed to r-µ*g* was interpreted as an indication that tumor cells are driven towards a less aggressive growth behavior.

#### 4.8.2. Papillary and Anaplastic Thyroid Carcinoma

A study from 2024 [116] examined s-µ*g*-induced changes (2D-clinostat, 3 d-exposure) in the appearance and gene expression of two South Korean thyroid cancer cell lines (SNU-790 and SNU-80). The adherent SNU-790 papillary thyroid cancer cells were characterized as elongated, spindle-shaped epithelial cells, while the adherent SNU-80 anaplastic carcinoma cells were identified as polygonal epithelial cells with large, round nuclei. Spheroid formation of SNU-80 cells was visible after just 24 h under s-µ*g*, whereas it took five days for SNU-790 cells. A significantly higher proportion of s-µ*g*-induced differentially expressed genes (DEGs) was detected in SNU-790 than in SNU-80, as revealed by an array-based transcriptome analysis. In SNU-790, the DEGs are related to histone and microRNAs that the authors linked to proliferation, growth, and differentiation. In contrast, some DEGs in SNU-80 showed a relationship to hypoxia.

### 4.9. Prostate Cancer

Prostate cancer ranks as one of the most prevalent cancers found in men. Even though there have been considerable improvements in early diagnosis and therapies, advanced forms of the disease frequently show resistance to standard treatments [117].

The intricate nature of the tumor microenvironment presents challenges for conventional in vitro and in vivo models, which, despite providing valuable insights, often fail to reflect its complexities accurately. However, recent advancements in space biology and tissue engineering present new possibilities for investigating prostate cancer in a µ*g*-environment. µ*g* serves as a distinctive platform for disease modeling and the development of innovative therapeutic approaches, as it triggers significant alterations in cell growth, 3D structures, and physiological responses [118,119,120].

Both r-µ*g* experiences and those created in laboratory settings have proven to be effective methods for generating scaffold-free 3D structures. µ*g*-induced spheroid models in cancer research offer valuable insights into metastasis, ECM formation, and alterations in gene and protein expression patterns, which are crucial for drug development and testing. These models guide stem cell differentiation and enable the construction of tissue-like constructs suitable for regenerative medicine and radiobiology. Reliable production of organoids, multicellular spheroids, small blood vessels, and cartilage and bone constructs—each of which presents challenges in traditional static cultures—is now achievable using the platforms designed initially for space physiology research described in Section 3. A key advantage of this approach is the scaffold-free formation, which eliminates the limitations imposed by artificial scaffolds and reduces interference with subsequent investigations [31,121,122,123,124].

Research on prostate cancer cell lines, specifically PC-3, LNCaP, and DU-145, conducted in a µ*g*-setting, indicates that the cells quickly detach from their surface and develop MCS within a time frame of 24 to 72 h. These changes are associated with increased collagen production, reorganization of F-actin, and altered expression of genes related to the extracellular matrix, such as *COL1A1* and laminins, which differentiate spheroid populations from those in a monolayer configuration [125,126].

The signaling pathways that play a role in cancer advancement, particularly the PI3K/AKT/mTOR and VEGF pathways, are controlled differently in µ*g* spheroids. The production of VEGF usually diminishes, while there is an increase in the activation of PI3K/AKT/mTOR, which promotes growth and adaptations associated with angiogenesis. Additionally, inflammatory cytokines like IL-6 rise sharply during spheroid formation, suggesting a connection between mechanical unloading and pro-tumorigenic paracrine signaling [125,126].

This aligns with findings from another study involving PC-3 prostate cancer cells. Temporary exposure of PC-3 cells to s-µ*g* using an RPM led to the development of two separate types of cells: 3D MCS and an adherent monolayer (AD). The MCS exhibited improved viability and a gene expression profile associated with aggressive tumor characteristics, including increased expression of genes linked to cell survival and motility, such as *COL1A1*, *MSN*, *HIF1A*, *TUBB*, and *ACTB*. Moreover, pro-inflammatory cytokines IL-6 and IL-8 were elevated, indicating their involvement in the initial stages of tumor clustering and suggesting them as potential therapeutic targets in metastasis. Overall, s-µ*g* has been established as an effective method for creating a scaffold-free model that better mimics the tumor microenvironment for studying cancer progression [94]. In terms of molecular signaling dynamics over the long-term, prolonged exposure to s-µ*g* (5 d on the RPM) resulted in a significant upregulation of *ERK1/2* expression in both AD and MCS cells, along with increased levels of *FN1* and *VCL1*, indicating a context of promoted tumor progression and mechanical adaptation [125].

In another study using the DU145 prostate cancer cell line, cultivated in a High Aspect Rotating-Wall Vessel (HARV), the 3D model displayed a phenotype that was generally less aggressive, less proliferative, and more differentiated compared to that observed in 2D cultures. This was linked to an imbalance in phospholipase metabolism and modifications in the PIK3 pathway [127].

A different analysis examined the spheroid formation of well-differentiated LNCaP cells alongside poorly differentiated DU145 cells in two separate culture conditions: mixed suspension culture (HARV) and static liquid-overlay plates. The DU145 spheroids exhibited greater sensitivity to alterations in composition and structure when cultivated in a mixed suspension environment than the LNCaP spheroids. About 40% of the DU145 cells in the mixed spheroids responded positively to the cell division marker Ki-67, whereas no positive results for this marker were observed in any of the LNCaP cells. This discrepancy suggests that the cells exhibit unique growth patterns not observed in static culture [128].

**Figure 5:**
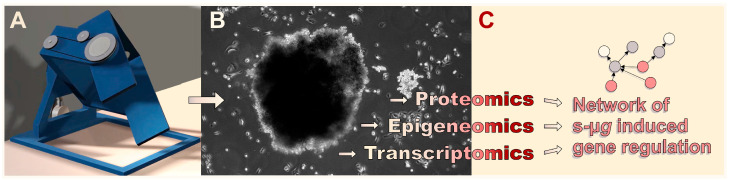
Ideally, multi-omics-based analysis of the effects of s-µ*g*-induced (**A**) changes in carcinoma cell physiology and (**B**) 3D organization leads to (**C**) the identification of causative interconnections of the gene response to the stimulus. (**B**) Here, the MCS of the prostatic adenocarcinoma cell line PC-3 after three days of RPM s-µ*g*-exposure is shown.

A recent study explores the differences in effects between µ*g* and normal gravity on prostate cancer cells. The findings suggest that 3D MCS models play a crucial role in identifying potential markers associated with tumor progression and therapeutic targets, particularly with respect to fibronectin, collagens, matrix metalloproteinases, and various signaling molecules. By employing a multi-omics strategy, the study assessed PC-3 prostate cancer cells that were exposed to s-µ*g* for 3 d (Figure 5). Notable findings revealed the rapid formation of 3D MCS, which are linked to the activation of genes regulating the cell cycle, blood vessel formation, and the structural organization of the ECM. Significant enrichment was found in gene ontology analyses in categories related to the extracellular environment and cellular interactions with a focus on the integrins, fibronectin network, and various forms of collagen in protein interactions. The research identified more than 11,000 differentially methylated positions, in addition to a significant differentially methylated region (DMR) in the SRMS gene, indicating substantial epigenetic alterations resulting from microgravity. Moreover, five s-µ*g*-related positive enrichments of transcription factor binding sites for AR, IRF1, IRF2, STAT1, STAT2, and FOXJ3 close to the DMPs were found. Genes associated with the ECM, cytoskeletal structure, and essential cytokines exhibited alterations revealed by transcriptional analyses. More DEGs were measured in AD compared to MCSs (N = 751 genes) and 1 *g* control cells compared to MCSs (N = 662 genes). Among others, cell cycle, angiogenesis, and cell adhesion genes were significantly enriched in GO annotations. In conclusion, the research highlights the importance of acknowledging these modifications within the context of prostate cancer studies [129].

Regarding the early r-µ*g* observations, the first experiment involving prostate cancer cells in actual µ*g* took place during the Columbia Space Shuttle mission. Although this experiment was unfortunately incomplete, it qualitatively demonstrated the formation of large 3D structures [130].

Finally, another study conducted in r-µ*g* provided direct evidence of the rapid response of prostate cancer cells to µ*g* conditions. Specifically, PC-3 cells that experienced brief periods of r-µ*g* during parabolic flights exhibited an immediate transcriptional response to changes in gravitational conditions. Through RNA sequencing (RNAseq), researchers found 298 genes that respond to gravity in a series of parabolic flight experiments. Crucially, these genes demonstrated significant functional enrichment in cytokines and chemokines, particularly with respect to key elements such as *IL6*, *CCL2*, *CXCL1*, *CCL20*, and *CXCL8*. These findings highlight the PC-3 cell transcriptome’s increased sensitivity, implying that signaling molecules and inflammatory factors are among the first to respond to gravitational changes. The study concluded that the stress induced by r-µ*g* rapidly activates pathways associated with PC cell signaling and tumor promotion [131].

In summary, studies examining prostate cancer cells in µ*g*-environments indicate that this distinct setting facilitates the natural creation of 3D spheroids without any scaffolds. They provide a model that better mimics physiological conditions for examining tumor behavior, metastasis, and responses to treatments. Significant molecular, structural, and epigenetic changes have been demonstrated to result from both r- and s-µ*g*, suggesting that it may be a novel approach to enhance cancer modeling and advance treatment.

### 4.10. Gastrointestinal Tumors

Several previous studies have shown that s-µ*g* and r-µ*g* substantially reshape the 3D tissue-engineered cancer phenotype across gastric, colorectal, pancreatic, and hepatic cancer cells. These studies used a range of µ*g* platforms and tissue engineering formats (spheroids, organoids, RCCS bioreactors, clinostat/simulated microgravity devices, and harmonized space-flown transcriptomics) to probe how altered gravity affects cancer cell morphology, growth, metabolism, gene expression, stemness, and drug response.

#### 4.10.1. Gastric Cancer

HGC-27 (human gastric cancer) cell metabolism under s-µ*g* (RCCS bioreactor) showed distinct alterations in key metabolic pathways, primarily involving glycerophospholipid and fatty acid metabolism. S-µ*g* led to the upregulation of phosphatidylethanolamine, phosphatidylcholine, arachidonic acid, and sphinganine, whereas sphingomyelin, phosphatidylserine, phosphatidic acid, L-proline, creatine, pantothenic acid, oxidized glutathione, adenosine diphosphate (ADP), and adenosine triphosphate (ATP) were downregulated [132]. Arun et al. [133] exposed gastric cancer stem cells (CSCs) and HCT116 colon cancer cells to a Rotary Cell Culture System (RCCS). The results revealed that the proportion of CSCs co-expressing CD44 and CD133 increased, and µ*g* influenced key regulators of growth and differentiation, including the PTEN/FOXO3/AKT pathway. Data also showed that µ*g* enhanced autophagy and led to a higher number of giant cancer cells exhibiting complete nuclear localization of Yes-Associated Protein (YAP). Rembialkowka et al. [134] reported that s-µ*g* (RCCS) could enhance the chemotherapeutic effects in gastric cancer models. This study demonstrated that s-µ*g*, especially when combined with doxorubicin, increased cytotoxicity in both drug-sensitive and resistant gastric cancer cells (EPG85-257 P and RDB). S-µ*g* reduced multidrug resistance gene expression, increased DNA/RNA damage markers, and caused major F-actin cytoskeletal reorganization, suggesting enhanced chemotherapy sensitivity under altered gravity conditions.

#### 4.10.2. Colorectal Cancer

Kim et al. [135] demonstrated distinctive growth and drug responses in colorectal cancer organoids under s-µ*g*. Their study used patient-derived colorectal cancer organoids and 3D clinostat organoids to examine transcriptomic and drug response changes under s-µ*g*. Organoids showed changes in morphology, increased viability, and upregulation of TBC1D3 genes and cell cycle pathways. Drug screening revealed enhanced sensitivity to 5-FU, indicating potential therapeutic implications of s-µ*g*-conditions.

#### 4.10.3. Pancreatic Cancer

Prolonged s-µ*g* promotes stemness, alters morphology, metabolism, and migration in pancreatic cancer, supported by proteomic/lipidomic/transcriptomic profiling. Massani et al. [136] reported that long-term s-µ*g* (RPM) altered pancreatic cancer cell morphology by activating Rho/Cdc42-mediated actin remodeling, promoted 3D spheroid formation and epithelial-to-mesenchymal transition, and activated the ERK5/NF-κB/IL-8 axis, expanding migratory cancer stem cells. S-µ*g* also induced HIF-1α/PI3K-Akt-driven metabolic reprogramming, which led to enhanced glycolysis and lipid synthesis to support survival under energy stress.

Pagano et al. [137] demonstrated that Trichostatin A (TSA) treatment under s-µ*g* suppressed pancreatic cancer cell proliferation, migration, and adhesion by downregulating key cell cycle, cytoskeletal, and adhesion proteins. This broad inhibition of pathways is essential for motility, stress response, and cytoskeletal integrity. The results of the study suggested that TSA under s-µ*g* may effectively impede tumor growth and metastasis. Separate work from Zeger et al. [138] demonstrated that pancreatic islets (PIs) survived well and exhibited strong beta-cell proliferation after short-term exposure (6 min) to r-µ*g* (MASER 15 sounding rocket experiment), especially in free-floating conditions. Even two weeks after exposure, the beta-cell proliferation persisted in 3D-printed islets. This suggested that space (r-µ*g*) conditions could stimulate beta-cell growth and viability, potentially enhancing islet regeneration or transplantation outcomes.

#### 4.10.4. Hepatocellular/Liver System

Cell-cycle regulator perturbations influenced by microgravity were also described in liver cell studies. Hoang et al. [139] established that s-µ*g* inhibited Chang liver cell proliferation by downregulating key cell-cycle regulators, including cyclins A and D and cyclin-dependent kinase 6 (Cdk6). S-µ*g* also altered the cytoskeleton by reducing the expression of actin and tubulin, components crucial for cell division and structure, leading to impaired cell proliferation.

Furthermore, Tuğcu et al. [140] demonstrated that HepG2 liver cancer cells formed viable 3D spheroids (>80% viability for 10 d) in both RCCS (Rotary Cell Culture System) and CSTR (Continuous Stirred-tank Reactor) bioreactors without scaffolds. The RCCS produced better-shaped spheroids, while CSTR provided better oxygenation and slightly higher viability. Urea production was similar (8.1 nmol/well in CSTR vs. 9.5 nmol/well in RCCS). Albumin output was initially higher in RCCS, but by day 8, CSTR surpassed it. Overall, both bioreactors effectively supported scalable 3D HepG2 culture.

Taken together, it can be concluded that gastrointestinal cancer cells are sensitive to both mechanical unloading and to culture system design. The harmonization of transcriptomic data from the different platforms is essential for a better understanding of the effect of µ*g* on the gastrointestinal system.

Table 4 provides a short summary of all µ*g* tissue engineering results with cancer cells.

## 5. Discussion

In general, tissue engineering techniques in s-µ*g* offer some considerable advantages over approaches in r-µ*g*. First and foremost, they are much cheaper and easily available. The ground-based facilities are relatively affordable, as there are various commercial providers, and they require no further specialized laboratory equipment going beyond what is typically used in biomedical and/or life science research. In addition, their operation is relatively straightforward and can be learned quickly. They enable a higher number of replicates and thus the detection of more subtle effects against the background as well as the production of larger amounts of engineered tissues. Lastly, in comparison to experiments in r-µ*g* in space, where delays and deviations from the protocol due to unforeseeable events are not uncommon, researchers have more direct control over the experimental conditions [141].

s-µ*g* devices have been used successfully with a multitude of cell types, both normal and malignant, and they are an important tool for the preparation of spaceflight missions. It was also shown in different experiments that s-µ*g* induces comparable effects to r-µ*g* in different cell types. Ingram et al. [142] demonstrated that prostate cancer cells formed MCS after HARV-exposure. MCS formation could be also confirmed in space during the STS-107 mission. After three days, pictures and data transmitted to Earth showed cancers the size of golf balls [130]. Moreover, similar results were found when thyroid cancer cells were grown in a scaffold-free culture on an RPM or in space. They revealed changes in their growth behavior [143,144]. Cells in the 1 *g*-inflight centrifuge grew as a 2D monolayer, as did the static control cells on Earth. 2D monolayers (adherent cells) and 3D MCS were observed in the spaceflight cell containers and in the RPM samples. Interestingly, the size of the MCS grown in space ranged from 5 to 10 mm in diameter, while RPM-MCS were smaller (2–3 mm) [144]. In addition, comparable findings were obtained when endothelial cells were exposed to an RPM or to space flight conditions. MCS and tube formation was found on both the RPM and in space [66,145].

However, ground-based facilities like the RPM, RWV, or clinostats can only simulate certain aspects of µ*g*. The biggest difference is the presence of fluid flow and shear forces. The movement of the culture vessels induces fluid flow inside them, which in turn manifests itself as shear forces acting on the cells. In addition, it affects nutrient and waste/metabolite transport, which is solely driven by diffusion in r-µ*g*. Bubble formation due to cell metabolism is also more critical in s-µ*g*. While they do not interfere with the experiment in r-µ*g*, they may cause substantial mechanical stress to the cells in a dynamic culture scenario [114].

But, these phenomena are not necessarily negative for tissue engineering applications using s-µ*g.* In the case of cartilage, some residual forces seem to be beneficial for the formation of a functional, matured tissue, as both Freed et al. and Stamenkovic et al. observed a superior quality of cartilage produced in an RWV on Earth compared to cartilage grown in LEO [146,147].

Overcoming these limitations might be difficult, as fundamental physical principles, such as the induction of flow inside a liquid-filled container upon movement, cannot be negated, regardless how elaborated the actual mechanical setup may be. There are currently endeavors to construct optimized devices with more fluid movements to induce less turbulence and algorithms to ensure equal exposure to similar levels of s-µ*g* on every position, but in the foreseeable future, current s-µ*g* tissue engineering techniques will not be able to replicate r-µ*g* conditions entirely. A first attempt using magnetic levitation in space has been successful [148]; however, it remains to be seen whether this technique is feasible under 1 *g* conditions on Earth.

## 6. Methods

**Figure 6:**
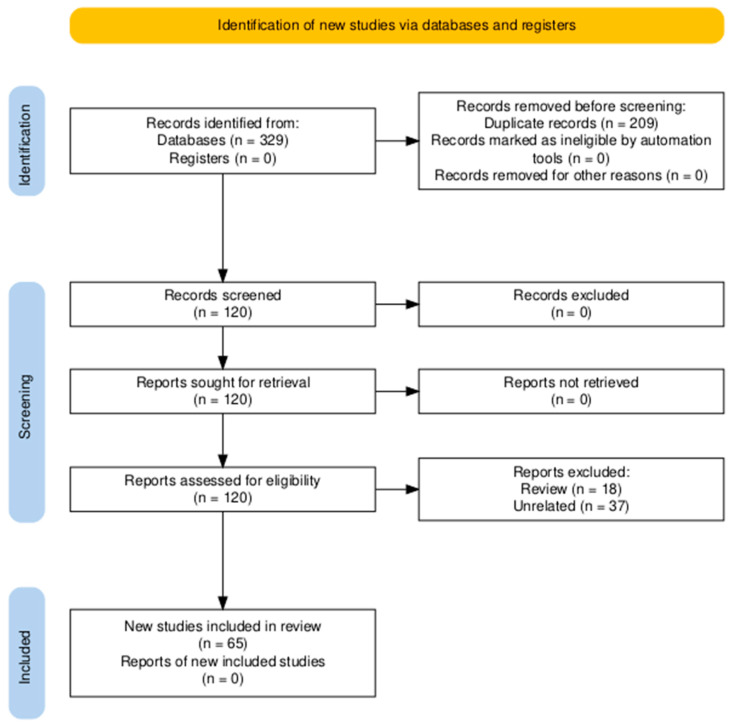
PRISMA flow diagram [149].

The PubMed database (https://pubmed.ncbi.nlm.nih.gov/, last accessed on 1 December 2025) was interrogated using permutations of the keywords “cartilage” or “chondrocytes” or “bone” or “tendon” or “vessels” or “endothelial cells” or “heart” or “cardiomyocytes” or “cardiac cells” or “breast cancer” or “lung cancer” or “thyroid cancer” or “prostate cancer” or “gastric cancer” or “colorectal cancer” or pancreatic cancer” or “liver cancer” or “hepatocellular cancer” with “microgravity” or “weightlessness” or “spaceflight”, and with “tissue engineering” or “spheroid” or “3D constructs” or “3d aggregates” or “organoid”. The search was confined to 2019–2025. Figure 6 displays the corresponding PRISMA flow diagram.

## 7. Conclusions

It has become clear that real and simulated microgravity conditions can clearly impact the field of tissue engineering [150]. Microgravity conditions can influence cellular biology, enabling the culture of 3D tissues without the structural limitations imposed by gravity. Significant advances in constructing spheroids and organoids from specialized cells or stem cells have been achieved [94].

Biofabrication in space offers important advantages for various research fields like pharmacology, biomedicine, translational regenerative medicine, and cancer research, among others. These advantages open new possibilities for drug development, for the reduction of the number of animal experiments, for tissue engineering, and for the design of novel biomaterials.

In this concise review, we have discussed the latest publications (2019–2025) about benign cells and various types of cancer cells studied under real and simulated microgravity, with focus on 3D spheroid or organoid formation. Biomanufacturing in orbit offers new opportunities to engineer organ tissues, supporting long-term human spaceflights and exploration adventures [151].

The ISS plays a key role in expanding this research. It provides a biofabrication lab, allowing researchers to study the impact of microgravity on different kinds of cells. The ongoing commercialization of spaceflight has already resulted in lower costs, increasing the possibility to perform space experiments and advance space biomanufacturing. The ISS National Lab has become an important center for the advancement of space sciences like fundamental sciences, in-space production application, technological development, manufacturing, and STEM education and workforce development. Cell research in space will be ongoing until 2030. Examples are projects like M4PM (Microgravity for Personalized Medicine)—Tumors, organoids, spheroids in space (ICE cubes Service, Space Applications, 1932 Sint-Stevens-Woluwe, (Brussels Area), Belgium), the future PRECISE (Prostate Cancer Cells in Space) CellBox-5 ISS experiment (YURI GmbH Meckenbeuren, Germany), neural organoids on the ISS [152,153], or iPSC-derived spheroids to be created in space [154,155]. The future will be experiments in LEO using other platforms, on the Chinese space station or on the Moon and the Mars in novel habitats, allowing us humans to live in outer space [156].

## Figures and Tables

**Table 1:** µ*g* platform overview used for tissue engineering applications.

	Space Station	Clinostat	Rotating Wall Vessel (RWV)	Random Positioning Machine (RPM)
**µ*g* quality**	10^−4^–10^−6^ *g*	Simulated	Simulated	Simulated
**Working principle**	Orbit	Gravity vector averaging by rotation about one axis	Gravity vector averaging by rotation about one axis	Gravity vector averaging by rotation about two axes
**Rotation speed**	NA	Depending on experiment 1–100 rpm	Adjusted, typ. 10–30 rpm	Typ. 10 rpm
**Experiment hardware**	Newly developed or reused flight certified hardware	Standard lab consumables or dedicated containers	Typ. dedicated containers	Standard lab consumables or dedicated containers
**Size limitation**	Size and weight are major cost drivers	Diameter of maximum a few millimeters	Typ. small suspended or bead attached samples (millimeter or submillimeter size)	Typ. smaller than 10 cm
**Accessibility**	Low, very costly	Very good	Very good	Very good
**Upload conditions**	Hypergravity, vibrations, temperature might not be well controlled	NA	NA	NA
**Delays**	Launch scrub likely	Immediate	Immediate	Immediate
**Fluid dynamics**	Minimal fluid motion	Laminar	Laminar	Complex, laminar and/or turbulent
**Shear stresses**	Very low	Low after initial speed up	Low after initial speed up	Noticeable, depending on experiment design
**Radiation**	Ca. 100 times higher	Low, location dependent	Low, location dependent	Low, location dependent
**Human intervention**	Limited, very costly	No specific limitation	No specific limitation	No specific limitation
**Sample collection**	Typ. fixation and subsequent cold stowage	Immediate and flexible	Immediate and flexible	Immediate and flexible
**Sample return**	Hypergravity, vibrations, temperature might not be well controlled	NA	NA	NA

**Table 2:** Overview of the physiological tissue-related results from tissue engineering in µ*g*.

Tissue	Microgravity Platform	Results	Reference
**Cartilage**			
Primary human chondrocytes and C28/I2 cell line	RVW, 14 days	Scaffold-free 5 mm large C28/I2 cell constructs and 3 mm large primary chondrocyte spheroids	[49]
Primary human chondrocytes	RPM, 5–28 days	Scaffold-free Spheroids, 2 mm in diameter by day 28	[50]
Human bone marrow mesenchymal stem cells (female and male donors)	Parabolic flight; Falcon 20 shuttle by the National Research Council of Canada. 11 parabolas	Successful induction of cartilage-like tissue in type I collagen porous scaffolds engineered cartilage tissues responded to microgravity in a sex-dependent manner	[51]
Primary human meniscus fibrochondrocytes (female and male donors)	RCCS-4 bioreactor, 7 days	Cell-seeded meniscus constructs in porous collagen scaffolds Sex-specific transcriptional responses like an upregulation of key osteoarthritis markers	[52]
**Bone**			
human foetal osteroblast cells (hFOB 1.19)	RPM, 7 and 14 days	Scaffold-free After 14 days: spheroids exhibited morphological characteristics indicative of bone-specific tissue organization	[54]
**Vessels**			
EA.hy926 cells and human microvascular endothelial cells	RPM, 7 days, 14 days ESA-SPHEROIDS ISS Mission 12 days	Scaffold-free 3D tubular constructs and 3D spheroids in Space and on the RPM	[64]
EA.hy926 cells	ESA-SPHEROIDS ISS Mission, 12 days	Scaffold-free 3D intima constructs Elevated IL-6 and IL-8 are involved in 3D formation	[66]
EA.hy926 cells	3D clinostat, 14 days, low and high glucose (HG)	Cells were viable and formed stable spheroids HG: number and size of aggregates increased Metabolic stress can enhance 3D morphogenesis	[67]
**Heart**			
Human induced pluripotent stem cell (hiPSC)-derived cardiac progenitors	ISS spaceflight, 3 weeks	3-fold larger sphere sizes, 20-fold higher counts of nuclei, and increased expression of proliferation markers. Improved Ca^2+^ handling and increased expression of contraction-associated genes.	[74]
Cardiac spheroids from hiPSC-CMs	ISS spaceflight, 8 days	Short-term ISS-exposure of 3D hiPSC-CMs induced altered i protein levels and gene expression changes (cell survival metabolism)	[76]

**Table 3:** Differences between classical tissue engineering and tumor engineering.

	Tissue Engineering	Tumor Engineering
Goal	Create biological substitutes to restore, maintain, or improve tissue function for medical applications.	Create models for understanding and combating cancer.
Methodology	Rebuild tissues outside the body before potential implantation.	Build complex, multi-cellular 3D models that mimic a patient’s tumor ex vivo.
Challenges	Finding a reliable cell source, and controlling cell proliferation, differentiation, and function to match the target tissue; recreating the correct 3D architecture and providing a supportive microenvironment for cell communication and tissue development	Recreate the full complexity of a tumor’s in vivo environment, including features like cellular heterogeneity, altered extracellular matrix (ECM), and the metabolic and inflammatory profiles of cancer cells.

**Table 4:** Overview of the cancer-related results from tissue-engineering in µ*g*.

Cancer	Microgravity Platform	Results	Reference
**Breast cancer**			
MCF-7 MDA-MB-231	2D-clinostat, 7 days	3D bio-printed MCS in hydrogel result in a stiffer ECM, influencing protein and gene expression levels, which could be modulated and sometimes even reversed in µ*g*	[95]
MCF-7 MDA-MB-231	RPM, 14 days	Scaffold-free MCS formation Positive association between the real metastatic microtumor environment and MCSs regarding ECM, cytoskeleton, morphology, and different signaling pathways, among others	[96]
MCF-7 MDA-MB-231	RPM, 24 h	Scaffold-free MCS formation BCL9, MYC, and JUN of the Wnt/β-catenin signaling pathway were differentially expressed in RPM-exposed MCF-7 cells Vinculin and β-catenin are key mediators to form MCS in µ*g*	[97]
CRL2351	RPM, 5 days	Scaffold-free 3D-culture model Substantial changes in cytoskeleton morphology, cytoskeleton-related gene and protein expression.	[98]
**Lung cancer**			
A549 human lung adenocarcinoma cells	ClinoStar™ system, up to 25 days	Scaffold-free NSCLC mini-tumor model The spheroids survived for 25 days and had a significant increase in growth	[103]
Squamous non-small-cell lung cancer cells (CRL-5889)	RPM, 4 days	Scaffold-free 3D spheroid formation Cytoskeletal changes Increased apoptosis after 24 and after 96 h µ*g* Upregulation of tumor suppressor genes (TP53, PTEN, RB1, and CDKN2A) and SOX2 compared to 1 *g*	[107]
Squamous non-small-cell lung cancer cells (CRL-5889) and Calu-3 adenocarcinoma cells	RPM, 3 days	Scaffold-free 3D	[108]
**Thyroid Cancer**			
FTC-133 follicular thyroid cancer cells	RPM, 4 h and 3 days	Spheroid formation is mediated by complex Wnt/β-catenin and TGF-β-related signaling	[110]
FTC-133, WRO and ML-1 cancer cells and Nthy-ori 3-1 thyrocytes	RPM, 3 days, dexamethasone	Mechanical stress influences this metastasis model system, processed differently by metastatic and healthy cells. The balance between adhesion, anti-adhesion, and cell–cell connections enables detachment of adherent cells on the RPM, or not, allowing selective inhibition of thyroid cancer in vitro metastasis by dexamethasone.	[113]
FTC-133 cells	RPM, 3 days	Formation of 3D spheroids is a two-step process: 1. detachment and 2. aggregation The RPM simulates physiological shear forces on the adherent cell layer. It offers a unique combination of environmental conditions for in vitro cancer research.	[114]
FTC-133 cells	ISS spaceflight, 5 and 10 days	Spheroid formation in space without any scaffolds. The response to microgravity was mainly anti-proliferative. ERK/RELA was identified as a major microgravity regulatory pathway	[115]
**Prostate cancer**			
PC-3 cells	RPM, 3 and 5 days	Spheroid formation, cytoskeletal alterations, deposition of collagen in the MCS Significant upregulation of genes belonging to the PAM pathway	[125]
PC-3 cells	RPM, up to 24 h	Spheroid formation, differential expression of the cytokines IL-1α, IL-1β, IL-6, and IL-8	[126]
PC-3 cells	RPM, 3 days	Spheroid formation; at a 5% FDR significance level, 11,090 genome-wide differentially methylated positions (DMPs) and one differentially methylated region in the SRMS gene in the 1 *g* vs. AD comparison, as well as an additional 10,797 DMPs in the 1 *g* vs. MCSs comparison.	[129]
**Gastrointestinal tumors**			
Human colorectal cancer (CRC) organoids	3D clinostat, up to 10 days	Significant dysregulation in the TBC1D3 gene family, increased viability, changes in cell cycle regulation Drug screening results indicated an enhanced response rate to 5-FU	[135]
Human ductal pancreatic adenocarcinoma cell lines PaCa-44 and CFPAC-1	RPM, 1, 7 and 9 days	Formation of 3D spheroids and enhancement of epithelial-to-mesenchymal transition The RPM activates ERK5/NF-κB/IL-8 axis remediate energy stress and apoptosis activation Metabolic reprogramming orchestrated by HIF-1α and PI3K/Akt pathways that upregulate glycolysis and impair β-oxidation, de novo synthesis of triglycerides for the membrane lipid bilayer formation	[136]
HepG2 cells, human liver carcinoma cell line	RCCS (Rotary Cell Culture System) and CSTR (Continuous Stirred-tank Reactor)	3D spheroids: (>80% viability for 10 days) in both RCCS and CSTR (Continuous Stirred-tank Reactor) bioreactors without scaffolds	[140]

## Data Availability

No new data were created or analyzed in this study. Data sharing is not applicable to this article.
